# Efficacy of a semirigid ankle brace in reducing mechanical ankle instability evaluated by 3D stress-MRI

**DOI:** 10.1186/s13018-021-02750-6

**Published:** 2021-10-18

**Authors:** Helge Eberbach, Dominic Gehring, Thomas Lange, Spartak Ovsepyan, Albert Gollhofer, Hagen Schmal, Markus Wenning

**Affiliations:** 1 Department of Orthopedic and Trauma Surgery, University Medical Center, Faculty of Medicine, University of Freiburg, Hugstetter Str. 55, 79106 Freiburg, Germany; 2 Department of Sport and Sport Science, University of Freiburg, Schwarzwaldstrasse 175, 79117 Freiburg, Germany; 3 Center for Diagnostic and Therapeutic Radiology, Medical Physics, University Medical Center, Faculty of Medicine, University of Freiburg, Kilianstrasse 5, 79106 Freiburg, Germany; 4 Department of Orthopaedic Surgery, Odense University Hospital, J.B. Winslows Vej 4, 5000 Odense, Denmark

**Keywords:** Mechanical ankle instability, 3D stress-MRI, Cartilage contact area, Ankle brace

## Abstract

**Background:**

Novel imaging technologies like 3D stress-MRI of the ankle allow a quantification of the mechanical instability contributing to chronic ankle instability. In the present study, we have tested the efficacy of a semirigid ankle brace on joint congruency in a plantarflexion/supination position with and without load.

**Methods:**

In this controlled observational study of *n* = 25 patients suffering from mechanical ankle instability, a custom-built ankle arthrometer implementing a novel 3D-stress MRI technique was used to evaluate the stabilizing effect of an ankle brace. Three parameters of joint congruency (i.e., 3D cartilage contact area fibulotalar, tibiotalar horizontal and tibiotalar vertical) were measured. The loss of cartilage contact area from neutral position to a position combined of 40° of plantarflexion and 30° of supination without and with axial load of 200 N was calculated. A semirigid ankle brace was applied in plantarflexion/supination to evaluate its effect on joint congruence. Furthermore, the perceived stability of the brace during a hopping task was analyzed using visual analogue scale (VAS).

**Results:**

The application of a semirigid brace led to an increase in cartilage contact area (CCA) when the foot was placed in plantarflexion and supination. This effect was visible for all three compartments of the upper ankle joint (*P* < 0.001; *η*^2^ = 0.54). The effect of axial loading did not result in significant differences. The subjective stability provided by the brace (VAS 7.6/10) did not correlate to the magnitude of the improvement of the overall joint congruency.

**Conclusions:**

The stabilizing effect of the semirigid ankle brace can be verified using 3D stress-MRI. Providing better joint congruency with an ankle brace may reduce peak loads at certain areas of the talus, which possibly cause osteochondral or degenerative lesions. However, the perceived stability provided by the brace does not seem to reflect into the mechanical effect of the brace.

*Trial registration* The study protocol was prospectively registered at the German Clinical Trials Register (#DRKS00016356).

## Background

Chronic ankle instability (CAI) arises from the two etiologies of functional (FAI) and mechanical ankle instability (MAI), whose interaction is the subject of ongoing research. These insufficiencies may result in perceived instability, generally presented by recurrent sprains or feelings of “giving way” [1, 2].

While functional insufficiencies should be treated by functional, e.g., sensorimotor training, mechanical deficits may require a mechanical intervention like an external support using an ankle brace, taping or even surgical stabilization [3, 4]. Ankle brace or tape is widely used in athletic populations in order to reduce recurrence rate or severity of sprains [3, 5]. The effectiveness of external supports lies in the limitation of joint excursion which reduces maximum inversion angle and angular velocity [6, 7]. These effects are accompanied by functional adaptations, e.g., the preparatory muscle activation, which is increased when wearing an external support [6, 8]. Due to this interaction leading to a combined neuromechanical effect, it is still subject to debate whether the amount of stabilization provided by ankle braces under load exceeds the effect of the active, neuromuscular stabilization [9, 10]. A measurement of the isolated mechanical deficit and its potential improvement by an ankle brace will help to estimate the effect of this common treatment. A systematic review found that the reduction of recurrent sprains may be independent of the type of external support, taping or bracing [11]. Furthermore, imaging studies have visualized the effect of ankle braces using different modalities like stress-roentgenology, arthrometric testing or, more recently, computed tomography [12–14].

However, diagnosing an unstable ankle is still challenging and has its limitations and flaws in practice, in particular when it comes to quantifying mechanical instability [1, 15]. A potentially useful parameter is the size of the contact area between the ankle’s cartilage surfaces as a three-dimensional correlate of joint congruency [16, 17]. The higher their congruency, the more stable the joint [18]. The measurable congruency changes during lateral opening, but has not been investigated much in connection with instabilities [19].

Novel imaging technologies like 3D stress-MRI of the ankle (3SAM) allow for a quantification of the mechanical instability contributing to CAI [17]. The three-dimensional joint congruency between the distal fibula and the talus may be a decisive factor in the development of perceived and mechanical instability [20]. This methodology also allows to investigate joint congruency with and without axial loading; thus, it may allow to estimate the stabilizing effect of an ankle brace on the different parts of the talocrural joint in vivo. Current evidence has shown that the main stabilizing effect will be in the vertical plane, while motion in the sagittal plane could be unrestricted [9, 21].

In the present study, we have tested the efficacy of an ankle brace in reducing the previously established measures of mechanical ankle instability using 3D stress-MRI. The aim of this study was to quantify the improvement of joint congruency achievable by wearing a semi-rigid ankle brace with and without axial loading in vivo. We hypothesized that an effective brace will have its main effect on the fibulotalar articulation while having a smaller effect on the horizontal tibiotalar articulation.

## Methods

The study was approved by the ethics committee of the University Medical Center of Freiburg (protocol #118/19), and the study protocol was prospectively registered at the German Clinical Trials Register (#DRKS00016356). It was carried out according to the Declaration of Helsinki in its current form, and all patients declared informed consent prior to participation.

### Population

This is a separate study on a subgroup of MAI patients deducted from a previous investigation [20]. The patients were recruited as a random community sample from the local university and outpatients of the university hospital’s orthopedic department.

Selection criteria were defined according to the literature sing the Cumberland Ankle Instability Score (CAIT) for defining perceived instability [15]. CAIT adds up a maximum score of 30 and any score < 24 is generally considered as a decisive criterion for CAI [22]. Mechanical instability was assessed by physical examination (talar-tilt and anterior drawer test) by a blinded experienced orthopedic surgeon [23]. For diagnosing mechanical ankle instability, both physical exams were rated in five steps (1 = stable, 2 = rather stable, 3 = intermediate, 4 = rather unstable, 5 = unstable) where there had to be a combined score > 8 in order to be rated as mechanically unstable. Moreover, an athletic background with an average sportive activity > 4 h per week was required. Exclusion criteria were previous surgery around the upper ankle joint, less than 3 months since the last ankle sprain and any contraindications to MRI diagnostics (tattoos, ferromagnetic implants) and acute illness.

Screening of *n* = 41 participants with subjective feelings of instability resulted in *n* = 25 included patients complying with the before-mentioned criteria. Reasons for the exclusion of screened participants were *n* = 6 presenting with perceived but no mechanical instability and *n* = 8 with intermediate scoring in CAIT or physical examination, *n* = 1 due to a novel tattoo in the region of interest and *n* = 1 due to a severe injury before final MRI examination. The final cohort of *n* = 25 patients showed average values of 24.6 ± 4.7 years of age, an average BMI (kg/m^2^) of 23 ± 3.5 and an average CAIT-Score of 18.8 ± 4.4.

### Mechanical testing

The mechanical stability testing was carried out using the previously described method of dynamic 3D stress ankle-MRI (3SAM) [17]. In this novel approach, the patient is placed supine in a custom-designed, non-ferromagnetic ankle arthrometer which allows free positioning of the foot in plantarflexion–dorsiflexion as well as pronation–supination. Furthermore, the device allows for the application of axial load up to 500 N using a pneumatic cylinder system. For axial loading, the patient is fixed to the table using a weightlifter's belt around the hip and adjustable straps tied to the table. In this study, the patients were measured under five different conditions as displayed in Table 1. The foot was therefore positioned neutral (NN) and in 40° of plantarflexion and 30° of supination (PS) without and with axial load of 200 N. The load was chosen from previous studies, where 200 N was the maximum load tolerated by the patients during PS-measurement without display of any adversities. To assess the effect of the ankle brace, it was worn in PS without and with axial load. The semirigid brace (MalleoLoc®, Bauerfeind AG, Zeulenroda, Germany) was composed of a plastic splint that was attached to the medial and lateral side of the ankle joint with two hook-and-loop straps assembled in a figure of eight.

**Table 1:** Measured conditions in 3D stress-MRI

Condition	Position	Load (200 N)	Brace	Rationale
1	NN	−	−	Individual reference value
2	PS	−	−	“Baseline” reduction of CCA
3	PS	−	+	Effect of brace
4	PS	+	−	Effect of load
5	PS	+	+	Effect of brace under load

NN = Neutral-null position, PS = plantarflexion–supination, CCA = cartilage contact area

All MRI experiments were performed on a Magnetom Trio 3 T system (Siemens Healthineers, Erlangen, Germany), using an 8-channel multipurpose coil (NORAS MRI Products, Germany) for signal reception. The protocol consisted of a 3D turbo-spin echo (TSE) sequence with GRAPPA parallel imaging acceleration by a factor of 2. The 3D imaging volume consisted of 128 sagittal slices with an in-plane resolution of 0.5 mm and a slice thickness of 0.6 mm.

In the post-processing, three different parameters of ankle joint congruity were calculated: cartilage contact area (CCA) in the fibulotalar (CCA_FT_) as well as the horizontal (CCA_TTH_) and the vertical (CCA_TTV_) part of the tibiotalar joint. The outcome parameters consisted of the individual reduction of CCA during plantarflexion-supination as a percentage of CCA in neutral-null position (s. Table 1, “Individual reference value”). Especially the loss of the CCA_FT_ has been shown to be a potential measure of mechanical ankle instability and its diagnostic strength is comparable to stress-sonography [17, 20]. Thus, a significant reduction of the loss in CCA, so-to-speak improvement of the joint congruency can be interpreted as a positive protective effect of the brace.

For post-processing of the MRI data, a browser-based framework for medical image analysis (Nora Medical Imaging Platform, Freiburg, Germany) was used.

### Patient-reported outcomes

Apart from the CAIT-Score, we used visual analogue scales (VAS) ranging from 0 to 10 to evaluate the perceived stability and comfort experienced by the participants in order to compare the subjective with the objective mechanical stabilization effect of the brace. The patients performed 10 lateral skater hops with and without wearing the brace before answering the VAS questions [24].

### Statistics

Statistical analysis was conducted using the Statistical Package for the Social Sciences (SPSS) version 27 (IBM Corp., Armonk, NY, USA). Graphical display was performed using Veusz (v. 3.0.1 by Jeremy Sanders).

For statistical comparison, after checking for normal distribution using the Shapiro–Wilk test, a two-factor repeated-measures ANOVA was carried out with the factors load and brace. Furthermore, the effect of the interaction load * brace was analyzed. The level of significance was set at *P* < 0.05. In cases of statistical significance, pairwise comparison was performed using Bonferroni-corrected t tests. Additionally, partial eta squared (*η*^2^) was calculated as a measure of effect size. Effect sizes were interpreted following Cohen (small: 0.01, medium: 0.06 and large: 0.12.) [25].

Moreover, bivariate two-tailed Spearman’s correlation analyses were conducted to determine the strength of the linear relationship between the difference in CCA resulting from the brace condition and the visual analogue scales. Correlation strength was interpreted according to Cohen as follows: < 0.3: weak correlation, > 0.3–0.5: moderate correlation, > 0.5: strong correlation [25]. Values are presented as mean values ± standard deviations.

## Results

**Fig. 1:**
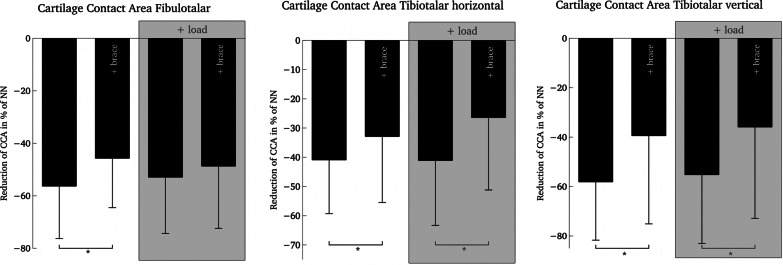
Relative changes in cartilage contact area with respect to the neutral-null position. CCA: cartilage contact area, NN: neutral-null position. Applied load: 200 Newton. *P < .05 in pairwise comparison

The results are displayed in Fig. 1 and Table 2. There was a significant effect of the brace (s. Table 2) on all three measures of CCA with the largest effect size on the horizontal tibiotalar CCA_TTH_ (*P* < 0.001; *η*^2^ = 0.54) in rm-ANOVA. The average loss of CCA was 10.6% less for CCA_FT_, 8.0% less for CCA_TTH_ and 18.7% for CCA_TTV_ compared to the no-brace condition.

**Table 2:** Cartilage contact areas (CCA) during plantarflexion-supination (40°/30°)

Parameter	No brace	w/ brace	No brace	w/ brace	rm-ANOVA factor load	rm-ANOVA factor brace
	At rest		+ 200 N load			
CCA_FT_	− 56.3 (20.0)	− 45.7 (18.8)	− 52.9 (21.4)	− 48.7 (23.7)	*P* = 0.71 *η*^2^ = 0.006	*P* = 0.004 *η*^2^ = 0.31
CCA_TTH_	− 40.9 (18.4)	− 32.9 (22.6)	− 41.1 (22.2)	− 26.4 (24.8)	*P* = 0.069 *η*^2^ = 0.13	*P* < 0.001 *η*^2^ = 0.54
CCA_TTV_	− 58.1 (23.6)	− 39.4 (35.7)	− 55.2 (27.8)	− 35.9 (37.0)	*P* = 0.4 *η*^2^ = 0.03	*P* = 0.002 *η*^2^ = 0.36

CCA_FT_ = fibulotalar articulation, CCA_TTH_ = tibiotalar horizontal articulation, CCA_TTV_ = tibiotalar vertical articulation, w/ brace: with brace, in brackets: SD

The effect of load when analyzed using rm-ANOVA did not result in significant differences (s. Table 2). Neither did the interaction effect of load*brace show significant results with *P* = 0.06 for CCA_FT_, *P* = 0.2 for CCA_TTH_ and *P* = 0.9 for CCA_TTV_.

Pairwise comparison revealed that there is a significant effect on CCA_TTH_ (*P* < 0.001) and a significant effect on CCA_TTV_ (*P* = 0.02) of the brace under load. However, there was no significant difference between the brace and no-brace condition under load in CCA_FT_ (*P* = 0.24).

**Fig. 2:**
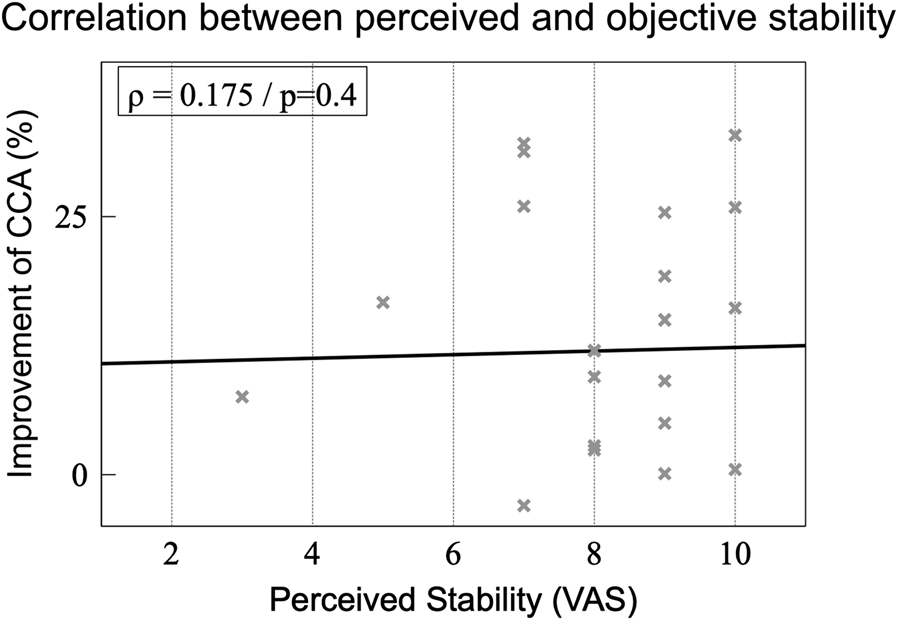
Correlation between perceived and mechanical instability displayed using 3SAM: CCA_FT_

Correlation analysis revealed that there were no significant correlations between the improvement of CCA when wearing a brace and the perceived stability provided by the brace. There was a significant correlation between the perceived stability (VAS 7.9) and the comfort (VAS 5.0) provided by the brace (Spearman’s rho = 0.48, *P* = 0.016). Furthermore, we evaluated the correlation between the perceived instability using VAS during the lateral Skater hop (VAS 7.9) and the quantitative improvement of the CCA_FT_ by the orthosis (10.6% of CCA), which did not correlate significantly (Spearman’s rho = 0.175, *P* = 0.4) (Fig. 2).

## Discussion

In this controlled observational study, we could demonstrate that semirigid ankle bracing has a significant effect on talocrural joint congruency in patients with mechanical ankle instability evaluated by 3D stress-MRI. More specifically, the application of a semirigid brace led to greater cartilage contact areas in a joint position close to maximum plantarflexion and supination.

Stabilizing the position close to full plantarflexion and supination is challenging for the whole ankle joint complex, especially in patients with mechanical ankle instability, generally associated with an insufficiency of the lateral ligaments of the ankle [26, 27]. In MAI patients, these structures do not sufficiently restrict anterior talar movement and posterior fibular slide, leading to a reduction in chondral and osseous constraint [28, 29]. A recent systematic review and meta-analysis showed that wearing an ankle brace can positively influence the kinematics of the foot and the ankle [3]. Mechanically, ankle braces have proven their ability to restrict vertical range of motion while allowing sagittal motion and this has been proposed to reduce the chance of further supination injury moments [30].

In the pilot study of Wenning et al., especially the reduction of CCA fibulotalar (corresponding to the lateral osseous constraint) in plantarflexion-supination was found to be decisive for the ankle's instability comparing healthy and mechanical instability patients [17]. We therefore hypothesized that an effective brace will have its main effect on the fibulotalar articulation.

Interestingly, in this study pairwise comparison showed that there was a significant effect of the brace only without load for fibulotalar CCA. This might be related to the fact that loading itself already increases the stabilizing capacities of articular surfaces. As an example, it has been shown that the articular congruency may increase when weight bearing after a lateral ankle fracture, but it may be transferred similarly to a sprain condition [31]. Additionally, early studies by Stormont et al. investigated the stabilizing capacity of the ligaments and articular surfaces in the ankle during supination. They demonstrated that the stabilizing capacity of the articular surface increases significantly during supination as the axial compression loading was raised from 0 to 670 N. These findings indicate that the articular surfaces display the individuals' predisposition and stability against supination with increase in axial compression [18]. The data of Tohyama et al. additionally showed that the contribution of bracing to stabilization of the ankle is dependent on the axial compression load across the ankle, suggesting that axial compression should be applied in the controlled condition in evaluation of ankle stabilizing devices. This means that the contribution of bracing to stabilization of the ankle was smaller in the axial loading condition than in the no axial loading condition [32]. When comparing these findings to our results, no significant effect of loading itself on cartilage contact areas was observed. Furthermore, the loss of articular surface during supination was less when wearing a brace; however, this beneficial effect was less pronounced when applying the axial load. This might be due to the custom-designed, non-ferromagnetic ankle arthrometer which only allows minimal motion and therefore also no significant change of contact areas during loading. Furthermore, it needs to be considered that the load applied in this study was a lot less (200 vs. 670 N), in order to enable the patient to keep the leg stable and without any adversities. This may also have reduced the effect of the loading in this study, which is why the results should be interpreted with care.

The findings match the assumption that external supports in preventing an ankle sprain may be pronounced mainly before ground contact because ankle bracing already reduces joint excursion during the swing phase of the gait cycle [33]. Alterations during the gait cycle in CAI like a decreased foot clearance partially due to increased plantarflexion and inversion angle may be two of the factors that can be improved by ankle bracing as a secondary preventive measure, since they reduce recurrent sprains [11, 34]. In this particular aspect, the preventive measure on recurrent sprains of a brace would come into effect during the swing phase or just before ground contact while the brace is not loaded.

Nevertheless, this study found a significant effect of the semirigid ankle brace on CCA_TTH_ and on CCA_TTV_ in pairwise comparison under load. This leads to an increase in the stabilizing capacity of the horizontal and vertical tibiotalar articular surface and more symmetric load distribution. Consequently, peak loads at certain areas of the talus possibly causing degenerative or osteochondral lesions may be reduced by the brace [16, 35]. When looking at these findings in detail, the increase in CCA_TTH_ will lead to a bigger surface for the distribution of weight and force during ground contact; subsequently, it would reduce the stress on the lateral and medial talar shoulder and prevent excessive impact on the cartilage and subchondral surfaces.

Moreover, the finding that the medial cartilage surface is also increased by the application of the brace may suggest that this increase joint congruency of the tibiotalar joint as a whole. However, the high variability of this parameter (CCA_TTV_) requires a careful interpretation of this finding. Additionally, from a detailed biomechanical point of view it needs to be discussed, that the increase on the tibiotalar joints is not matched by an increase in the fibulotalar CCA, which can only be realized when there is a certain amount of joint play in the tibiofibular joint. Evidently, this is an early explorative aspect of this investigation and it requires further research including a dynamic analysis that can differentiate between tibiotalar, fibulotalar and tibiofibular biomechanics. Especially the latter is a challenge for research, which is why the role of the anterior inferior tibiofibular ligament in lateral ankle instability remains a mystery. Even though it is indicative, that braces have a tertiary preventive effect on the degeneration of the joint resulting from maldistribution of load, it cannot be concluded from these findings, but it should be the focus of future, longitudinal studies.

The interaction of the different components in CAI is part of an ongoing debate and we therefore included subjective measures as a potential correlate to mechanical performance of the brace. However, in our study there was no significant correlation between the improvement of CCA_FT_ when wearing a brace and the perceived stability provided by the brace. This supports the hypothesis that mechanical and perceived insufficiencies are distinct entities contributing to chronic ankle instability [36]. Furthermore, it promotes the theory that the functional, e.g., sensorimotor improvement, is a major factor in the effectiveness of ankle bracing [37]. Again, additional research is necessary to further examine the relationship between functional and mechanical instabilities of the ankle to thoroughly evaluate the different adjustments effectuated by the brace.

Moreover, this underlines the recommendation that diagnosis of ankle instability with radiographic measurements alone is imprecise, because MAI can occur without subjective instability of the ankle and vice versa [8, 19]. In the future, it should be examined, which percentage in loss of CCA is clinically meaningful and these current findings of ankle efficacy will need to be compared to that. It may then also be of interest to compare conservative and operative procedures and their impact on CCA.

### Limitations

This study has a few limitations to consider, among which is the static testing of the cartilage contact areas. Therefore, transferring our results to the highly dynamic landing during sports performance should be made cautiously. Nevertheless, we were testing the benefit in of the brace in a close to accident position, which is of great scientific interest. Testing in even more different flexion and supination angles with MRI would have been excessively time consuming. Another limitation is the relatively small sample size as it was a subgroup of MAI patients deducted from a previous investigation. Furthermore, it has to be considered that all measures were performed by a single investigator and except from the pilot study no inter-rater reliability can be reported. However, this novel method has been shown to be practical and robust for analyzing CAI before [20].

## Conclusion

In conclusion, we found a significant effect of the semirigid ankle brace on joint congruency verified by using 3D stress-MRI. This effect may reduce peak loads at certain cartilage areas of the ankle and possibly delay degenerative or osteochondral lesions.

## Data Availability

The data which analyzed during the study are stored in our hospital and are available from the corresponding author on reasonable request.

## Abbreviations

CAI: Chronic ankle instability
MAI: Mechanical ankle instability
FAI: Functional ankle instability
CAIT: Cumberland ankle instability tool
CCA: Cartilage contact area
ICC: Interclass coefficient
3SAM: 3-Dimensional stress ankle-MRI
